# Reduced Digestibility of Maize Starch in Freeze-Thaw Cycles: Mechanistic Insights into the Role of Fish Scale Peptides

**DOI:** 10.3390/foods15213943

**Published:** 2026-11-03

**Authors:** Yuxue Zheng, Zhilong Wu, Yanming Ren, Xu Chen, Shaoyun Wang

**Affiliations:** 1 College of Biological Science and Engineering, Institute of Food and Marine Bio-Resources, Fuzhou University, Fuzhou 350108, China; 2 College of Food and Health, Zhejiang A&F University, No. Wusu Street 666#, Hangzhou 311300, China

**Keywords:** starch, freeze-thaw cycles, peptides, multi-scale structure, digestion

## Abstract

Freeze-thaw (FT) cycles cause structural deterioration of maize starch (MS), accelerating starch digestibility and glycemic responses. This study investigated whether crimson snapper fish scale peptides (FSP) could modulate MS structure to reduce digestibility during FT cycles. FSP inhibited ice recrystallization and preserved bound water while suppressing free water increase. After three FT cycles, amylose content in MS-8FSP remained at 24.17% while 28.17% in MS, while Mw was unchanged. Hydrophobic interactions dominated FSP-starch binding, though hydrogen bonds weakened during freezing. SEM, XRD and DSC showed that MS-8FSP formed compact lamellar networks, with relative crystallinity increasing to 5.29% and Peak II enthalpy recovered after three FT cycles. Consequently, FSP reduced rapidly digestible starch and estimated glycemic index (MS-8FSP: 69.22; MS: 92.06). Pearson correlation analysis revealed that FSP binding and non-covalent bonds were negatively correlated with rapidly digestible starch and estimated glycemic index, whereas water permeability was positively correlated with both. In vivo, MS-8FSP significantly attenuated postprandial glucose responses in mice, with lower AUC than MS across all FT cycles. These findings suggest that FSP enhances starch FT stability and reduces digestibility, offering a promising ingredient for frozen starch-based foods with reduced glycemic potential.

## 1. Introduction

Starch is the primary carbohydrate polymer in the human diet and a key determinant of postprandial glycemic responses [1,2]. Its digestion rate and extent depend not only on its multi-scale structure—including chain length distribution, crystalline type, and lamellar organization—but also on its interactions with other components in the food matrix [3,4]. However, during processing and frozen storage, starch-based foods inevitably undergo repeated freeze-thaw (FT) cycles. Ice crystal formation and recrystallization physically disrupt the starch gel network, increase granular fragility, and expose additional enzymatic cleavage sites, ultimately accelerating starch digestion and elevating glycemic responses [5,6]. Conventional strategies-adding sugars, salts, or hydrocolloids-often compromise palatability or introduce excess calories, and fail to preserve starch structural integrity under FT stress [7]. Hence, novel ingredients that simultaneously inhibit ice recrystallization and protect starch structure are highly desirable.

To address this, chemical [8], physical [9,10,11,12], and enzymatic modifications [13,14], have been developed, along with the addition of hydrophilic colloids [15,16], fiber [17], gums [18], and antifreeze proteins. Their core mechanisms include: inhibiting ice crystal formation and recrystallization [19], enhancing gel network strength [20,21], regulating water [22], and protecting crystalline structures [23,24]. However, chemical methods may introduce non-food-grade agents, physical methods are energy-intensive, and conventional additives often lack digestive stability benefits. Natural protectants with both cryoprotective and anti-digestive functions are urgently needed.

Antifreeze peptides are small protein hydrolysates that reduce ice crystal damage in sub-freezing environments [25,26], offering lower cost, better stability, and abundant availability from by-products [7]. They inhibit ice recrystallization by adsorbing onto ice-water interfaces, induce thermal hysteresis, and protect biological macromolecules against freezing [27]. Antifreeze peptides have been derived from plant and animal processing by-products, including zein and gelatin hydrolysates [28], gelatin hydrolysates, and wheat glutenin hydrolysates [29], sericin peptides [30]. Fish-derived bioactive peptides have attracted increasing attention as natural cryoprotectants due to their abundance from processing by-products. Among these, fish skin collagen peptides (FSCP) have been reported to improve the FT stability of starch-based dough systems. Duan et al. [31] demonstrated that FSCP (0–2.0%, *w*/*w*) inhibited water migration, reduced free water content, and decreased rapidly digestible starch content in frozen wheat dough, attributing the protective effect to a peptide layer formed on starch granule surfaces. Similarly, Antarctic krill peptide (AKP) has been shown to restrain water migration and interact with amylose in frozen dough, thereby preserving starch structural integrity [27]. These studies have established that marine-derived peptides can improve the FT stability of starch-based systems.

However, existing studies have primarily focused on macroscopic quality attributes such as water distribution, rheological properties, and starch digestibility measured after freezing, rather than on the underlying ice crystal behavior and the structural evolution of starch during repeated FT cycles. In particular, how fish-derived peptides regulate ice crystal growth and how the resulting structural changes in starch translate into altered digestion behavior remain poorly understood. Fish scale-derived peptides (FSP) from crimson snapper-a fishery by-product-are known for their antioxidant activity and processing stability [32]. However, their potential to suppress ice recrystallization in starch matrices and modify starch digestion during FT cycles remains unexplored.

To fill this gap, this study investigated whether FSP could modulate maize starch (MS) structure to improve FT stability and reduce digestibility. We systematically characterized the effects of FSP on water status, starch-peptide interactions, amylose content, molecular weight, crystalline structure, thermal stability, and in vitro digestive kinetics of MS under repeated FT cycles. Through multi-scale structural and functional analysis, we aimed to establish a mechanistic basis for FSP application in frozen starch-based foods, offering a strategy to address both quality and nutritional challenges in frozen carbohydrate polymer systems.

## 2. Materials and Methods

### 2.1. Materials

Maize starch was purchased from Jiwo Food Co., Ltd. (Zhengzhou, China), FSP was produced by our lab. The relevant information was as follows: The FSP was extracted by using ultrasonic-assisted high-pressure pretreatment and enzymatic hydrolysis, the detailed extraction method were shown in Supplementary Materials S1. The molecular weight of the extracted product was mainly concentrated below 3000 Da, accounting for 99.07% of the total enzymatic hydrolysis products. The content of hydrophobic amino acids was 29.91%, while the content of polar amino acids was 52.54%. Gly (19.20 g/100 g) and Pro (11.78 g/100 g) had the highest content. The extraction method, molecular weight distribution and amino acid composition were shown in Tables S1 and S2. Sucrose was obtained from Angel Yeast Co., Ltd. (Yichang, China), α-amylase from *Bacillus subtilis* and pepsin was obtained from Yuanye Bio-Technology Co., Ltd. (Shanghai, China), α-glucosidase from *Aspergillus* sp. was obtained from SAITONG Biotechnology Co., Ltd. (Guangzhou, China). A GOPOD-format glucose assay kit was purchased from Megazyme International (Bray, Co., Wicklow, Ireland). All other reagents were of analytical grade.

### 2.2. Ice Recrystallization Inhibition (IRI) Assay

The IRI activity was assessed following Jiang’s protocol with minor modifications [33]. Sample solutions were prepared at 5 mg/mL in 10% (*w*/*v*) sucrose, rapidly frozen at −30 °C for 5 min to achieve complete solidification, and subsequently held at −6 °C for 20 min. During this incubation, images of ice crystals were recorded at 5, 10, and 25 min using a CCD camera (Nikon D-7500, Tokyo, Japan). Images were analyzed with ImageJ (Version 1.54, Bethesda, MD, USA), after threshold adjustment and calibration to calculate the ice crystal area. All IRI measurements were performed with three independent replicates (*n* = 3).

### 2.3. Preparation of MS-FSP Complexes

Base on the results of the pre-experiment, starch samples were dispersed in deionized water (5%, *w*/*v*) with FSP added at 0%, 2%, 4%, 6%, 8%, and 10% (*w*/*w* starch), stirred at room temperature for 5 min, then heated at 95 °C for 30 min with continuous stirring to fully gelatinize. The cooled gels were divided into 0, 1, and 3 FT cycles groups. Each sample was frozen at −18 °C for 12 h and thawed at 25 °C for 12 h per cycle. And followed by vacuum freeze-dried. The resulting samples were ground and sieved (100-mesh) to obtain the MS-FSP complexes. In the sample naming, “MS” represents maize starch, and the numbers preceding “FSP” indicated the addition concentration of FSP. 0, 1 and 3 represented the number of cycles.

### 2.4. FT Ability

As decribed in Section 2.3, the cooled gels were divided into 0, 1, and 3 FT cycles groups. Each sample was weighed (M_1_), frozen at −18 °C for 12 h and thawed at 25 °C for 12 h per cycle. After centrifugation at 3000 rpm for 20 min, released water was removed and weighed (M_2_). Water release rate was calculated as:

Permeability rate (%) = M_2_/M_1_ × 100%

where: M_1_ is the initial mass of the gel sample before FT; M_2_ is the free water after centrifugation. The permeability rate was measured with three independent replicates (*n* = 3).

### 2.5. Water Molecular Mobility

For freeze-thawed starch gels, the starch gels were subjected to 0, 1, 3 FT cycles (each cycle: freezing at −18 °C for 12 h, followed by thawing at 25 °C for 12 h). After the final thawing, the samples were equilibrated at room temperature for 10 min before being placed into nuclear magnetic resonance tubes. Subsequently, LF-NMR analyzer (VTMR20-010V-T; Niumag, Shanghai, China) and CPMG sequence (0.8 ms echo time, 5000 echoes, 4 scans) were used to measure the transverse relaxation time (T_2_) and amplitude (A_2_) [34]. LF-NMR measurements were performed with three independent replicates (*n* = 3).

### 2.6. Determination of Amylose Content and Molecular Weight of Starch

The amylose content of starch samples was determined using an amylose/amylopectin assay kit (Megazyme International, Bray, Ireland). The amylose content was calculated from the glucose released after enzymatic hydrolysis of the supernatant. The molecular weight distribution of starch was determined by high-performance size exclusion chromatography (HPSEC) on an Agilent 1100 system (Agilent Technologies, Santa Clara, CA, USA) coupled with multi-angle light scattering (MALS) detection. Starch samples (5 mg) were dissolved in DMSO/LiBr (0.5%, *w*/*v*) at 90 °C for 10 h, filtered through a 0.45 μm membrane, and separated on a GRAM Lux column (1000 Å, 8 × 300 mm, 10 μm; Agilent) at 60 °C with a flow rate of 0.5 mL/min. The weight-average molecular weight (Mw) was calculated using ASTRA software (Version 8.2, Wyatt Technology, Santa Barbara, CA, USA). Amylose content and Mw were determined with three independent replicates (*n* = 3).

### 2.7. Chemical Interaction Forces

The chemical interaction forces of the samples were determined according to the method described by Huang et al. with minor modifications [35]. Four reagents were prepared using 0.05 mol/L phosphate buffer (pH = 7.0), including 0.05 mol/L NaCl (N1), 0.6 mol/L NaCl (N2), 0.6 mol/L NaCl +1.5 mol/L urea (N3), and 0.6 mol/L NaCl +8 mol/L urea (N4). Each sample (100 mg) was mixed with 5.0 mL of the respective reagent. The mixture was then placed in a 37 °C water bath and shaken for 120 min, followed by centrifugation at 10,000 rpm for 10 min. Protein content in the supernatant was determined using the Coomassie Brilliant Blue G-250 method. FSP content was calculated using a standard curve prepared with bovine serum albumin as a reference. Differences in FSP content between N1 and N2, N2 and N3, N3 and N4 were attributed to changes in the concentrations of ionic bonds, hydrogen bonds, and hydrophobic interactions, respectively. The chemical interaction force measurements were performed with three independent replicates (*n* = 3).

### 2.8. X-Ray Diffraction (XRD)

XRD analysis was performed following Zheng et al. [36] with slight modifications. Freeze-dried samples were scanned using a Bruker D2 PHASER diffractometer (Karisruhe, Germany) (Cu Kα radiation, λ =1.542 Å) at 4–40° (2θ) with a scanning speed of 0.05°/s (40 kV, 40 mA). Relative crystallinity was calculated using MDI Jade 6.5. XRD measurements were performed with three independent replicates (*n* = 3).

### 2.9. SEM

The microstructure of the freeze-dried MS-FSP complexes after 0, 1, and 3 FT cycles was observed using a scanning electron microscope. Samples were mounted on aluminum stubs with double-sided conductive tape and sputter-coated with gold under vacuum. Images were acquired at an accelerating voltage of at ×500 magnification. For each sample, three independently prepared samples were observed, and representative regions were selected for imaging (*n* = 3).

### 2.10. DSC

Dried starch samples (~3 mg) were sealed in aluminum crucibles with deionized water (sample:water = 1:3, *w*/*w*) and equilibrated at room temperature for 10 h. DSC was performed under N_2_ (50 mL/min) from 20 to 120 °C at 10 °C/min, with an empty crucible as reference. Gelatinization parameters (To, Tp, Tc, ΔH) were determined from the thermograms. DSC measurements were performed with three independent replicates (*n* = 3).

### 2.11. In Vitro Digestion Properties of the Complexes

#### 2.11.1. In Vitro Simulated Digestion

In vitro digestion was conducted following Zhang et al. [37] and Zheng et al. [38] with modifications. Complexes (containing 500 mg starch) were dispersed in 5 mL water, gelatinized at 95 °C for 30 min, then equilibrated to 37 °C. Gastric digestion was initiated by adding 4 mL simulated gastric fluid (pH 3.0), 80 mg pepsin, and 25 μL 0.3 mol/L CaCl_2_, and incubated at 37 °C for 30 min. Then 5 mL acetate buffer (0.4 mol/L, pH 5.2) was added, followed by 5 mL simulated intestinal fluid (150 U/mL α-amylase, 1.3 U/mL amyloglucosidase, 0.3 mol/L CaCl_2_) for intestinal digestion at 37 °C for 180 min. Digestive solution (200 μL) were collected at 0, 15 and 30 min during the simulated gastric stage and at 0, 20, 30, 60, 90, 120 and 180 min during the simulated intestinal stage. Each aliquot was mixed with 1800 μL 80% ethanol, centrifuged at 10,000 rpm for 10 min. Glucose was measured using a glucose oxidase kit, and RDS, SDS, and RS contents were calculated as following equations:

RDS = (G_20_ − G_0_) × 0.9 × 100%

SDS = (G_120_ − G_20_) × 0.9 × 100%

RS = 100% − (RDS + SDS)

where G_0_, G_20_ and G_120_ were the glucose content in the sample after hydrolysis for 0, 20 and 120 min during the simulated intestinal stage, respectively.

Commercial white bread (Anjoy Foods Group Co., Ltd., Xiamen, China) was used as the reference food. The total starch content of the white bread was determined and matched to that of each test sample prior to hydrolysis. The white bread was subjected to exactly the same hydrolysis protocol as the test samples, and its area under the hydrolysis curve (AUC) was set as 100 for the calculation of hydrolysis index (HI). The hydrolysis curve was used to determine the hydrolysis index (HI), estimated glycemic index (eGI) as described by Granfeldt et al. [39] and subsequently adopted in Li et al. [40]:

HI = (AUCsample/AUCwhite bread) × 100

eGI = 0.862 × HI + 8.1981

Starch hydrolysis kinetics were modeled using a non-linear first-order equation:

Ct = C_ꝏ_ (1 − e^−kt^)

where: Ct is the hydrolysis percentage at time t, C_ꝏ_ is the equilibrium hydrolysis extent; k is the digestion rate constant. In vitro digestion experiments were performed with three independent replicates (*n* = 3).

#### 2.11.2. Determination of FSP Release

The amount of FSP released during digestion was determined according to the method described by Vilcacundo et al. [41]. Briefly, the digested fluid from the end-gastric (120 min) and end-intestinal (120 min) phases of the digestion process was boiled in a boiling water bath for 10 min, respectively. Subsequently, the solutions were centrifuged at 4000 r/min for 15 min to obtain the supernatant and precipitate, respectively. FSP content was calculated using a standard curve prepared with bovine serum albumin as a reference. FSP release measurements were performed with three independent replicates (*n* = 3).

### 2.12. Postprandial Glucose Responses

Male C57BL/6J mice (SPF, 6–8 weeks, *n* = 56) were housed under a 12 h light/dark cycle at 25 ± 2 °C with free access to water and diet. After one week of acclimation, mice were randomly divided into 7 groups (*n* = 8 per group) (FZU 2025-SG-020), comprising six starch sample groups (MS-0, MS-1, MS-3, MS-8FSP-0, MS-8FSP-1, MS-8FSP-3) and one glucose control group. Starchy samples (MS-0, MS-1, MS-3, MS-8FSP-0, MS-8FSP-1, MS-8FSP-3) were gelatinized (1.8 g in 18 mL water, 95 °C, 30 min) and cooled to 37 °C. Following 12 h fasting, blood glucose was measured by tail clipping using a glucometer. Mice received glucose solution (2 mg/g body weight, 200 mg/mL) or sample suspensions (0.1 mL/10 g body weight) by gavage. Blood glucose was measured at 0, 30, 60, 90, and 120 min post-administration, and the area under the curve (AUC) was calculated. All in vivo data were obtained from eight mice per group (*n* = 8).

### 2.13. Statistic Analysis

The results were expressed as mean ± standard deviation. Two-way analyses of variance were performed with SPSS 20.0 (SPSS Inc., Chicago, IL, USA) to determine the significance of differences between variable means at 0.05 levels. Experimental graphs were constructed using Origin 8.5 (OriginLab Corporation, Northampton, MA, USA).

## 3. Results and Discussion

### 3.1. Anti-Freeze Ability of the MS-FSP Complexes

#### 3.1.1. Ice Recrystallization Inhibition

**Figure 1:**
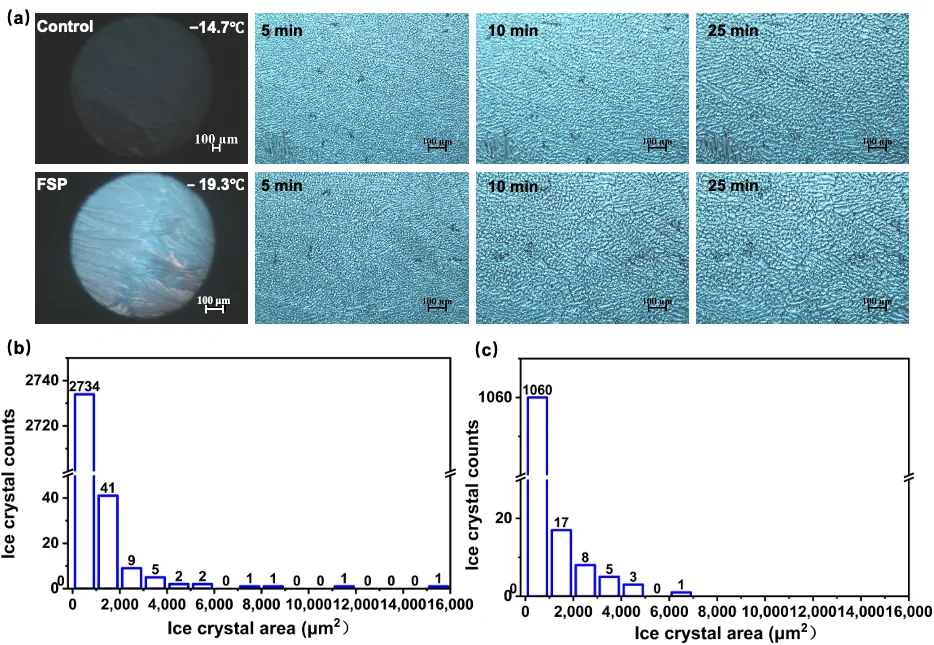
Ice recrystallization inhibition activity (**a**) and ice crystal size of control (**b**) and FSP (**c**) at 25 min.

Ice recrystallization inhibition was assessed by cold-stage microscopy, in which the onset temperature of the first observable ice crystal and the crystal size distribution after recrystallization were recorded and quantified with ImageJ. FSP increased the ice crystal onset temperature from −14.7 °C to −19.3 °C (Figure 1a), indicating that FSP interferes with the ordered arrangement of water molecules, raises the homogeneous nucleation barrier, and delays initial ice crystal formation [7]. It should be noted that the onset temperature measured by cold-stage microscopy reflects the first observable ice crystal in the field of view and is therefore used for relative comparison rather than as an absolute nucleation temperature. During recrystallization, the control group exhibited faster crystal growth and larger final sizes than the FSP group (Figure 1b,c). ImageJ quantification further showed that crystals in the control were predominantly <2000 μm^2^, whereas those in the FSP group were mostly <1000 μm^2^; crystals <500 μm^2^ accounted for 65.02% (control) and 86.37% (FSP), respectively. These results demonstrate that FSP effectively inhibits ice recrystallization and restricts crystal growth-a prerequisite for mitigating the mechanical damage that ice crystals impose on the starch gel network during FT cycles. This ice crystal-regulating capability provides the basis for the structural protection of starch discussed below [27].

#### 3.1.2. FT Permeability of the MS-FSP Complexes

**Figure 2:**
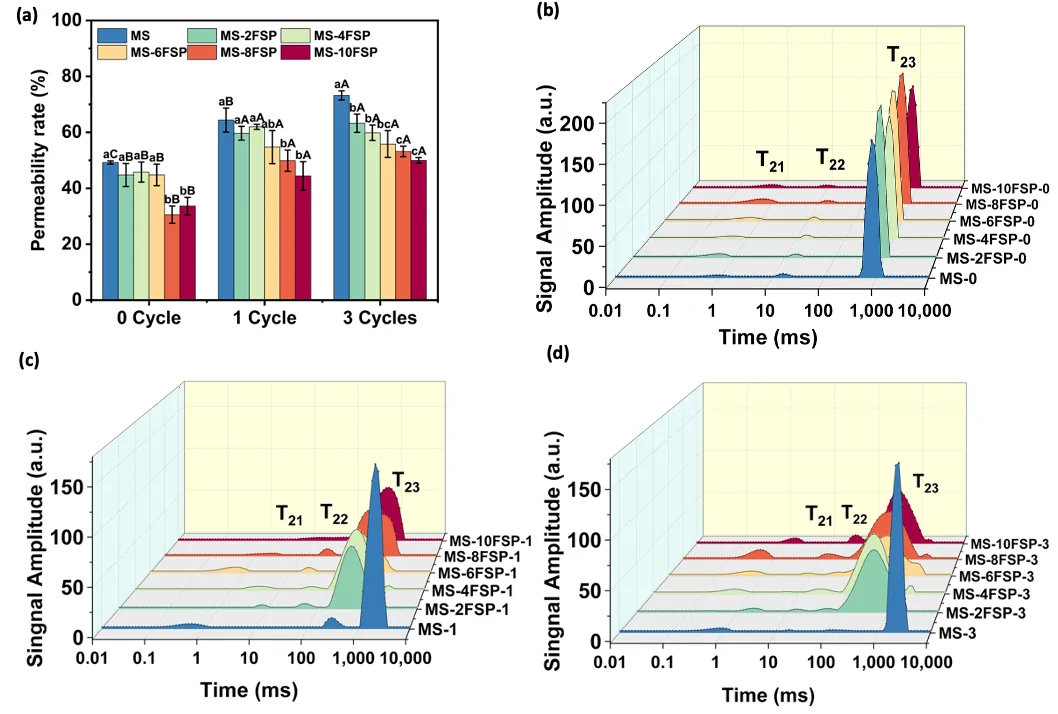
Permeability rate (%) (**a**) and LF-NMR patterns of the starchy samples during 0 (**b**), 1 (**c**) and 3 (**d**) FT cycles. Different lowercase letters indicated significant differences among different FSP addition levels, and different uppercase letters indicated significant differences among different FT cycles.

Freeze–thaw permeability was determined by measuring the extent of water release from the starch gels after repeated FT cycles, with higher values indicating poorer structural stability. Across 0 to 3 cycles, MS had the highest permeability and poorest stability (Figure 2a). Co-gelatinization with FSP effectively reduced permeability, with higher polypeptide amounts providing stronger inhibition of water release. Permeability increased with repeated cycles as ice crystals damaged the starch matrix, but high FSP addition (8% and 10%) alleviated this damage by forming hydrogen-bonded composite networks with starch [11,42]. MS-10FSP exhibited the least water release and the best FT stability. This supports Zhang et al., who reported that protein-polypeptide co-gelatinization fills pores from starch rehydration, weakens molecular rearrangement, and reduces ice crystal-induced water release [37]. Overall, FSP co-gelatinization improved both water retention and FT stability of starch systems.

#### 3.1.3. LF-NMR

Water distribution and mobility in the starch gels were characterized by LF-NMR, in which T_21_, T_22_, and T_23_ were assigned to tightly bound, moderately bound, and free water, respectively, and lower relaxation times indicate tighter water binding and greater stability. As shown in Figure 2c,d and Table 1, FSP significantly altered water distribution before FT (0 Cycle). In the MS control, T_23_ increased from 841.50 to 2235.1 ms and T_22_ from 22.0 to 139.8 ms over 0–3 FT cycles (*p* < 0.05), while T_21_ dropped sharply from 3.3 to 0.5 ms after 1 cycle, indicating freeze-induced gel network disruption and water redistribution. In contrast, FSP-treated groups exhibited significantly lower T_23_ and T_22_ than the control (*p* < 0.05). Notably, MS-4FSP and MS-6FSP maintained low T_23_ (418.8 and 506.9 ms) and T_22_ (31.3 and 14.1 ms) at 3 cycles, with no significant change from 0 cycle (*p* < 0.05), confirming that FSP effectively preserves the water-holding network during FT.

Different FSP concentrations showed distinct regulatory effects. At 0 cycle, all FSP groups had lower T_21_ (from 0.4 to 0.6 ms) than MS (3.3 ms) (*p* < 0.05), indicating tighter water binding. MS-2FSP exhibited a T_22_ rise to 321.2 ms at 3 cycles (*p* < 0.05), indicating insufficient protection at low concentration. In contrast, MS-4FSP, MS-6FSP, and MS-8FSP maintained relatively low T_22_ (31.3, 14.1, and 24.5 ms at 3 cycles, respectively) and T_23_ (396.2, 418.8, and 506.9 ms at 3 cycles, respectively), confirming that 4–8% FSP effectively preserved the water-holding network during FT cycles. However, MS-10FSP showed an abnormal T_21_ increase to 9.17 ms at 1 cycle (*p* < 0.05), suggesting excessive FSP weakened bound water stability after the 1 FT cycle.

### 3.2. Physicochemical Changes of MS-FSP Complexes During FT Cycles

#### 3.2.1. Binding Efficiency of FSP to Starch

The binding amount (BA) and loading rate (LR) of FSP on starch were determined by measuring the free FSP content in the supernatant after centrifugation. BA and LR reflect the efficiency of FSP binding to the starch matrix and its retention during FT cycles. At 0 cycle, BA increased proportionally with FSP concentration, rising from 16.19 mg/g at 2FSP to 81.65 mg/g at 10FSP (Table 2), indicating that the initial loading capacity depended on the amount of FSP available for complexation. During FT, BA declined in all samples, reflecting partial dissociation of FSP from the starch matrix. The MS-2FSP group showed the greatest loss, with BA decreasing from 16.19 to 11.83 mg/g and LR from 80.95% to 59.13%, suggesting that low FSP concentrations were insufficient to form stable complexes capable of withstanding ice crystal-induced mechanical stress. In contrast, MS-4FSP to MS-10FSP maintained higher BA and LR after 3 FT cycles, indicating that higher FSP concentrations favored the formation of more stable binding structures.

#### 3.2.2. Amylose Content and Mw of Starch During Freeze Thaw Cycles

Amylose content was determined using the Megazyme amylose/amylopectin assay kit, and Mw was measured by HPSEC-MALS. Amylose content and molecular weight are fundamental molecular parameters of starch that determine its crystalline organization and susceptibility to enzymatic hydrolysis. In the MS group without adding FSP, amylose content significantly increased from 20.67% in the 0 cycle to 28.17% in the 3 FT cycles (*p* < 0.05), indicating that the mechanical damage of ice crystals promoted the physical release of amylose from starch granules and gel networks; while after adding FSP, the upward trend of amylose content was significantly inhibited. The amylose content of MS-4FSP to MS-10FSP (23.83–24.50%) after 3 FT cycles were all lower than that of MS (28.17%), with MS-4FSP being the lowest (Table 2). These results showed that FSP can effectively protect the integrity of the starch structure and reduce the leaching of amylose during FT cycles.

Different from the significant change in amylose content, the Mw of all samples did not show significant changes during FT cycles, as the Mw of MS slightly decreased from 2.13 × 10^6^ g/mol in the 0 cycle to 2.01 × 10^6^ g/mol in the 3 cycles (*p* > 0.05), and there were no significant differences in Mw among the FSP-added groups at different cycle numbers (*p* > 0.05), indicating that although the mechanical force of ice crystals can damage starch granules and gel networks, it was not sufficient to break the covalent glycosidic bonds of starch molecules. The significant increase in amylose content and the non-significant decrease in M_w_ suggested that the damage of FT cycles to the starch structure was essentially a physical dissociation rather than chemical degradation.

#### 3.2.3. Micro-Structure of the Complexes

**Figure 3:**
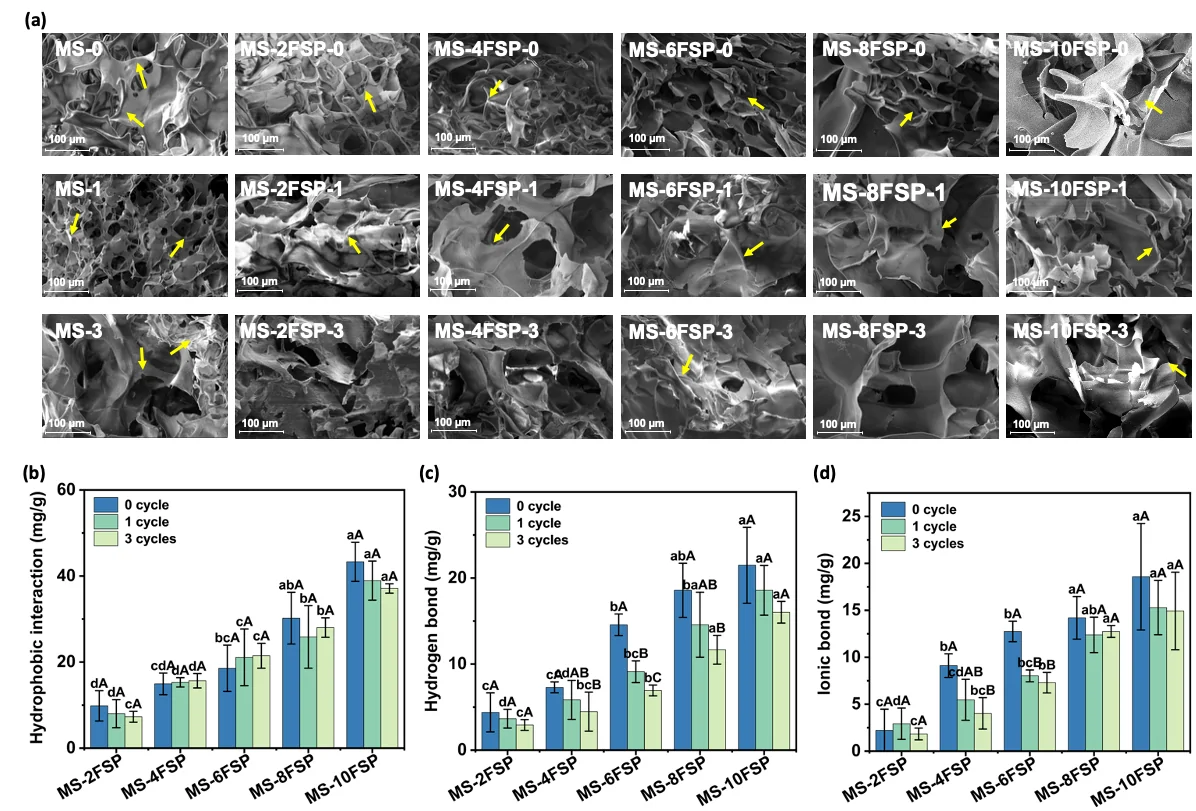
The effect of FSP on starch surface structure (**a**), ×500, scale bars 100 μm, chemical interaction forces of the complexes during 0 (**b**), 1 (**c**), 3 (**d**) FT cycles. The yellow arrows indicated pores. Different lowercase letters indicated significant differences among different FSP addition amounts, and different uppercase letters indicated significant differences among different FT cycles (*p* < 0.05).

SEM morphology observations provided evidence for the improvement of FT stability of FSP at the microstructure level (Figure 3a). In samples without FSP added, after 3 FT cycles, the starch framework presented a distinct linear filamentous morphology, with numerous irregular pores interspersed between the elongated starch strips, and the network structure was severely damaged—this characteristic corresponds to the significant increase in T_23_ in LF-NMR (an increase in free water filling the pores) and the decrease in network rigidity caused by hydrogen bond breakage [27]. After adding FSP, as the concentration increased from 2% to 10%, the starch morphology gradually changed from linear filamentous to sheet-like or layered structures, with the layer arrangement becoming more regular and the number of pores significantly reduced; the MS-10FSP group still maintained a dense and continuous sheet-like structure after 3 FT cycles. This densification trend is consistent with the research results of Li et al. [43].

#### 3.2.4. Chemical Interaction Forces

The chemical interaction forces were determined by sequentially treating the complexes with N1–N4 reagents and measuring the released FSP content in the supernatant. Upon increasing FSP from MS-2FSP to MS-10FSP, hydrophobic interactions, hydrogen bonds, and ionic bonds all increased, with contribution following the order: hydrophobic interaction > hydrogen bond > ionic bond (Figure 3b–d). The linear starch possesses a hydrophobic helical cavity that allows spontaneous insertion of hydrophobic amino acid side chains, serving as the core driving force; starch hydroxyl groups form hydrogen bonds with peptide polar groups to stabilize the complex, while the extremely low ionization degree of starch makes ionic interactions the weakest [16]. Higher peptide concentrations expose more hydrophobic residues, significantly strengthening hydrophobic association [44], whereas hydrogen and ionic bonds are limited by total functional groups and increase gradually.

#### 3.2.5. Long-Range Order of the MS-FSP Complexes

**Figure 4:**
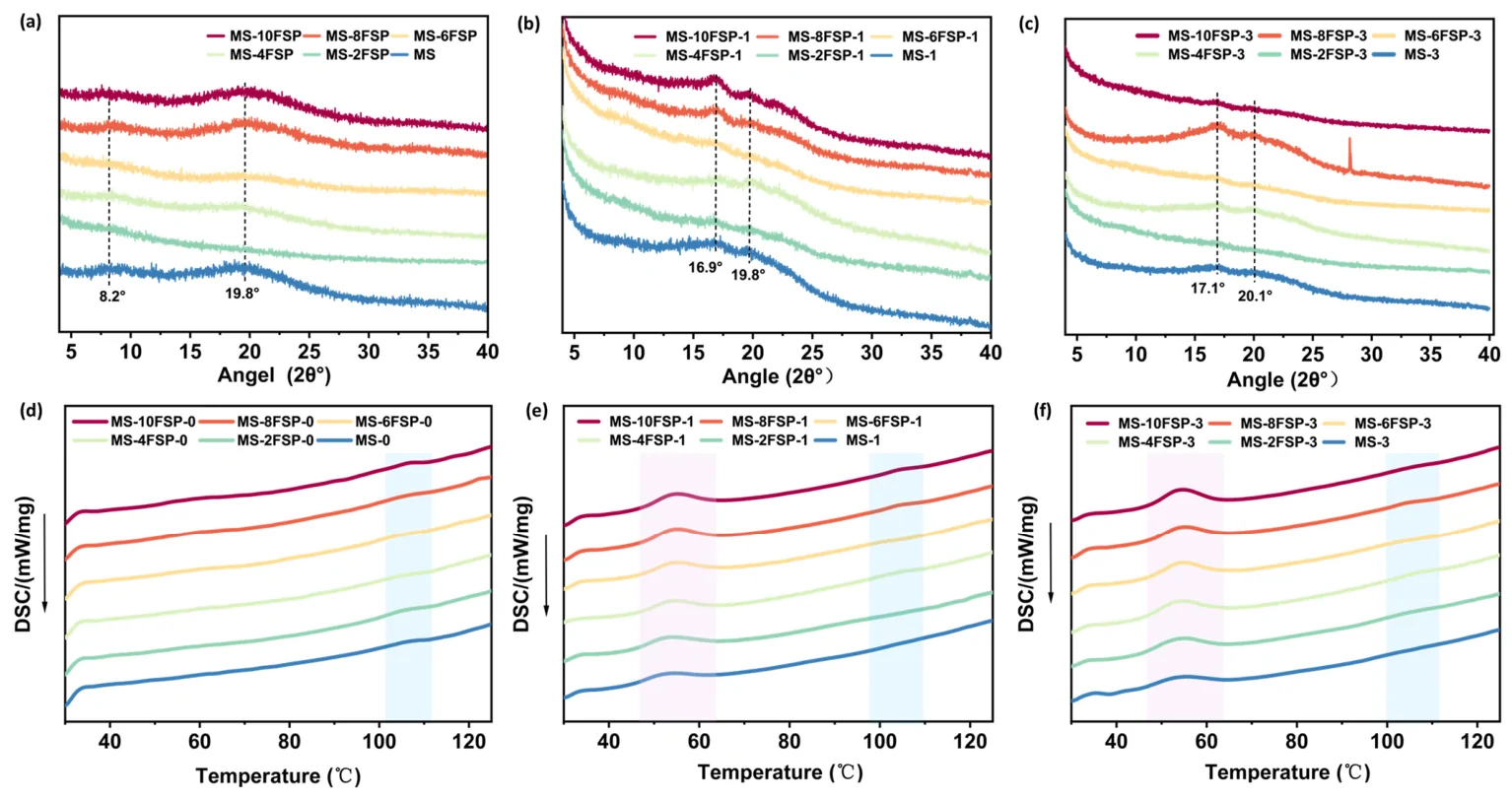
XRD patterns of the complexes treated with 0 (**a**), 1 (**b**) and 3 (**c**) FT cycles; DSC curve of the complexes treated with 0 (**d**), 1 (**e**) and 3 (**f**) FT cycles. The stripes in light pink and blue represented peak I and peak II.

Long-range ordered structure was characterized by XRD, and relative crystallinity was calculated using MDI Jade 6.5. RC of MS decreased from 5.13% to 2.37% after FT, indicating that ice crystal-induced crystalline disintegration [43]. MS-2FSP and MS-6FSP groups remained at very low RC levels (0–2.37%), MS-4FSP exhibited the most stable RC (from 4.36% to 4.01%), while MS-8FSP showed a unique V-type recovery (from 3.68% to 5.29%) (Table S1). XRD analysis revealed that after 1 cycle, all samples displayed diffraction peaks at 17.0° and 20° (Figure 4a), with intensities increasing at higher FSP concentrations, suggesting that FSP promoted ordered chain arrangement and formation of B- or V-type crystals (Figure 4b). After 3 FT cycles, MS-8FSP showed the strongest 17 ° Cpeak intensity (Figure 4c), consistent with its RC recovery to 5.29%, confirming that MS-8FSP induced cold crystallization or recrystallization during repeated FT [45]. However, this structurally favorable effect did not continue linearly at higher FSP concentrations. The absence of further improvement at 10FSP suggested that there is an optimal FSP concentration for promoting crystalline reorganization, beyond which additional polypeptide may become structurally ineffective or even disruptive. Possible reasons include saturation of FSP–starch interactions, steric hindrance and network formation by excess polypeptides, competition for water and hydrogen bonding, and phase separation or heterogeneous distribution of FSP within the starch matrix.

#### 3.2.6. DSC

Thermal properties of the MS-FSP complexes were characterized by DSC, in which the endothermic peak temperature and ΔH of each transition were recorded to reflect the dissociation of ordered structures and the rearrangement of starch chains. DSC analysis (Table 3, Figure 4d–f) showed that untreated MS-0 exhibited only a single endothermic peak (Peak II, about 95–110 °C), attributed to dissociation of V-type complexes formed by amylose-lipid/FSP hydrophobic segments. Peak II ΔH increased with FSP concentration, indicating enhanced formation of heat-resistant V-type ordered structures. After 1 FT cycle, a new low-temperature endotherm (Peak I, about 45–55 °C) appeared in all samples, attributed to amylose rearrangement and recrystallization following ice crystal-induced gel network disruption [17]. Peak I ΔH increased with FSP concentration after 1 FT cycle and further increased after 3 FT cycles, with greater increments than native starch, suggesting FSP promoted starch chain rearrangement and ordered stacking during FT.

Peak II ΔH decreased significantly after FT (MS from 0.44 to 0.04 J/g; MS-2FSP from 0.55 to 0.16 J/g), indicating mechanical disruption of V-type order by ice crystals. However, after 3 cycles, ΔH generally recovered, attributed to FSP’s dual effects: hydrophobic interactions driving new V-type helix formation and ice recrystallization inhibition applying mild concentration pressure to induce ordered stacking [27]. This destruction-reconstruction balance led to net enthalpy recovery, with FSP-added groups showing significantly higher recovery than pure starch, confirming FSP’s both cryoprotective and cold crystallization-inducing functions.

The DSC and XRD results represented the combined effects of starch structural evolution and FSP-starch interactions, and that the individual contribution of FSP content cannot be fully deconvoluted in the current experimental design. We have also noted this as a limitation and suggested that future work could include control experiments with FSP alone, physical mixtures of FSP and starch without gelatinization, and varying FSP concentrations to further clarify this point.

### 3.3. Digestive Behavior of the MS-FSP Complexes

**Figure 5:**
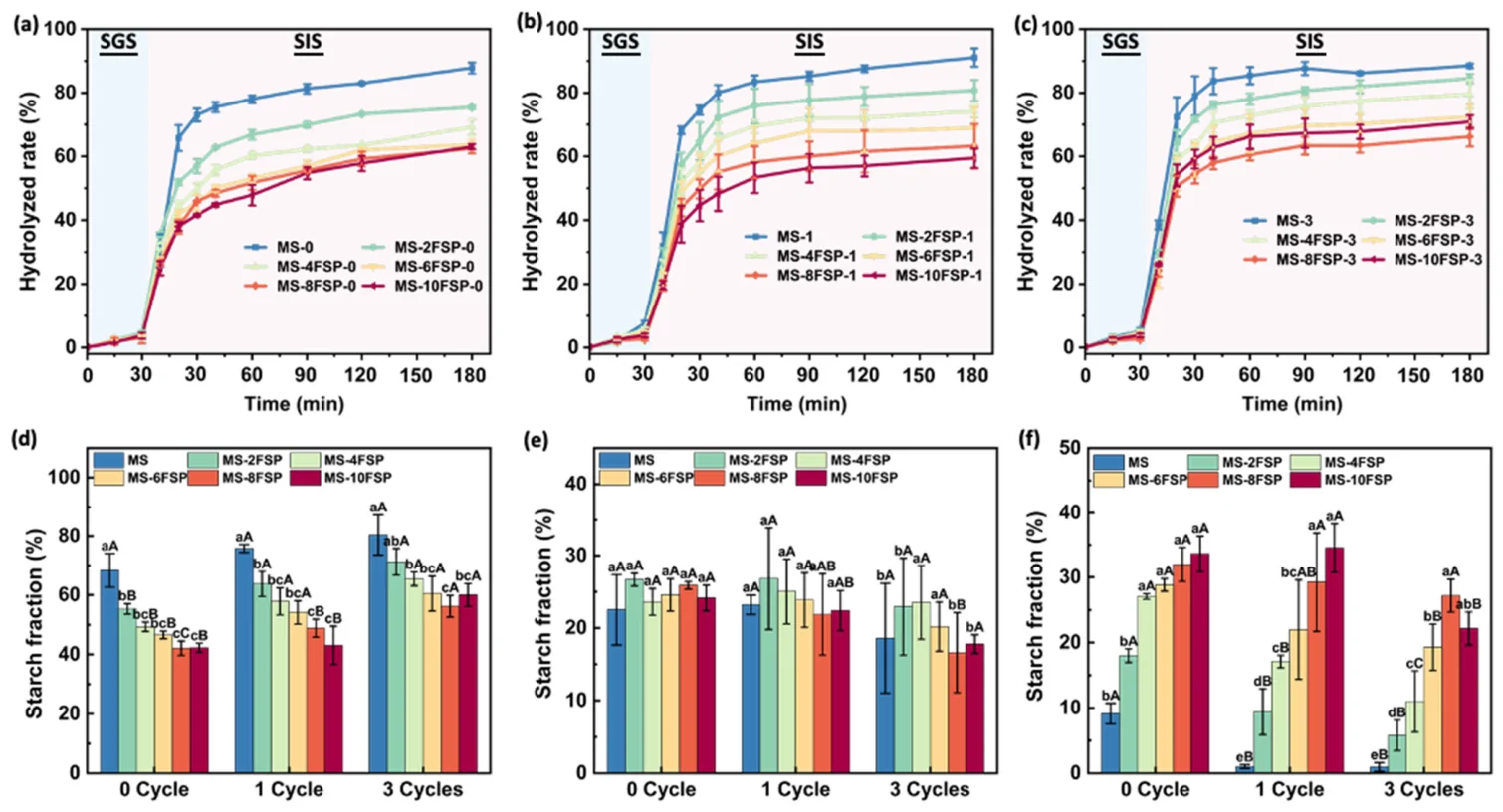
Digestive property of the MS-FSP complexes. Hydrolyzed curve of starchy samples treated with 0 (**a**), 1 (**b**) and 3 (**c**) FT cycle, RDS (**d**), SDS (**e**) and RS (**f**) content treated with 0, 1 and 3 FT cycles. Different lowercase letters indicated significant differences among different FSP addition levels, and different uppercase letters indicated significant differences among different FT cycles. SGS and SIS represented stimulated gastric stage and intestinal stage, respectively.

The digestive behavior of the MS-FSP complexes was evaluated by simulating gastrointestinal digestion in vitro, in which the samples were first subjected to pepsin under acidic gastric conditions and then to intestinal digestion, with the degree of hydrolysis monitored as a function of time and fitted to obtain the equilibrium hydrolysis extent (C∞, %). As shown in Figure 5a, MS-FSP complexes underwent only mild degradation during gastric digestion, primarily due to limited pepsin-induced hydrolysis of FSP under acidic conditions [46]. In contrast, hydrolysis efficiency increased markedly during intestinal digestion. With increasing FT cycles (1 and 3), the hydrolysis curves of all samples shifted upward, indicating enhanced digestion sensitivity after FT (Figure 5b,c) [27]. However, FSP-added groups consistently exhibited lower hydrolysis curves than MS, with a more pronounced downward trend at higher FSP concentrations (MS-2FSP to MS-10FSP). The equilibrium hydrolysis extent (C∞, %) declined continuously and stabilized (Table 4), confirming that FSP effectively suppressed the FT-induced increase in starch digestibility. Notably, MS-8FSP and MS-10FSP maintained relatively low hydrolysis levels after 3 cycles, further confirming that FSP preserved starch granule and gel network integrity, reducing enzyme accessibility.

Starch fractions were further analyzed to explore structural evolution during digestion (Figure 5d–f). At 0 cycle, MS showed the lowest RDS and highest RS contents, while FSP-added groups had slightly lower RS than MS, possibly due to FSP-starch interactions altering partial ordered structures. With increasing FT cycles, MS exhibited significantly increased RDS and decreased RS (*p* < 0.05), indicating that FT destroyed crystalline regions and double helices, converting enzyme-resistant ordered structures into loosely packed, readily digestible states. After FSP addition, RDS still increased with FT cycles but was significantly lower than MS at the same cycle (*p* < 0.05), with a more gradual RS decline. Previous studies have shown that Antarctic krill peptides have also been reported to delay starch recrystallization during freezing storage, protect granule integrity, and reduce digestibility [27]. This was consistent with the mechanism of FSP in this study, which reduced starch digestibility by inhibiting ice recrystallization and maintaining the integrity of the gel network. MS-8FSP retained relatively higher RS content after 3 FT cycles, corresponding to the lowest terminal hydrolysis rates. Similar concentration-dependent behavior has been reported for other additives, they found that 0.6% guar gum exhibited the highest storage and loss moduli across all FT cycles, whereas higher concentrations were less effective [47]. This parallels the present finding that 8% FSP approached the optimal concentration, beyond which no further structural or digestibility benefits were observed.

### 3.4. Postprandial Glucose Responses

Postprandial glycemic responses were evaluated in vivo by administering the selected samples and calculating the area under the blood glucose curve (AUC, mmol/L·h^−1^). Based on the in vitro digestion profiles and FT stability, the MS-8FSP was selected as the representative sample for in vivo glycemic response evaluation. For MS, the AUC values were 10.70 ± 1.26 (0 cycle), 11.38 ± 0.81 (1 cycle), and 11.89 ± 0.98 (3 cycles) mmol/L·h^−1^. In contrast, MS-8FSP gave significantly lower AUC (7.53 ± 1.01 with 0 cycle), 8.22 ± 0.85 (1 cycle), and 8.94 ± 0.69 (3 cycles) mmol/L·h^−1^. respectively (Table 5). Across all FT cycles, the AUC of MS-8FSP was 25–30% lower than that of MS, and the difference was statistically significant (*p* < 0.05). This reduction in AUC indicated that FSP incorporation effectively attenuated the postprandial glucose surge, consistent with the in vitro finding that FSP reduced the proportion of RDS by protecting starch granule integrity.

AUC increased with increasing FT cycles in both groups, but the increase was not statistically significant among 0, 1, and 3 FT cycles, suggesting that 3 FT cycles were insufficient to cause significant deterioration in glycemic response. Notably, the absolute AUC of MS-8FSP remained consistently lower than that of MS even after 3 FT cycles, further confirming that the glycemic-stabilizing effect of FSP was sustained throughout FT treatment.

**Table 5:** Postprandial blood glucose AUC value (mmol/L⋅h^−1^) in mice after starch administration.

Samples	0 Cycle	1 Cycle	3 Cycles
MS	10.70 ± 1.26 ^aA^	11.38 ± 0.81 ^aA^	11.89 ± 0.98 ^aA^
MS-8FSP	7.53 ± 1.01 ^bA^	8.22 ± 0.85 ^bA^	8.94 ± 0.69 ^bA^

Note: Different lowercase letters indicated significant differences among different FSP addition levels, and different uppercase letters indicated significant differences among different FT cycles (*p* < 0.05). The data were presented as the mean value ± SD, *n* = 8.

### 3.5. Pearson Correlation Analysis

**Figure 6:**
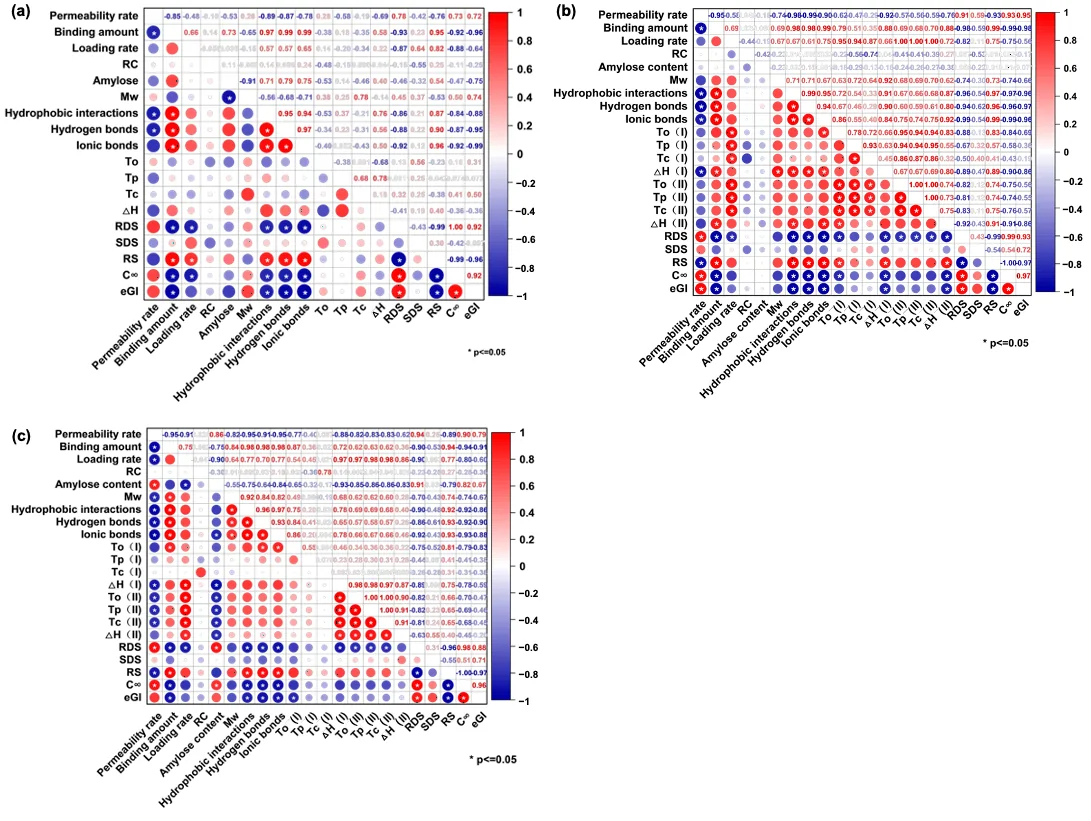
Correlation analysis of starch’s FT stability, structural characteristics and digestive properties under FSP intervention after 0 (**a**), 1 (**b**) and 3 (**c**) FT cycles.

Pearson correlation analysis was performed to evaluate the relationships among the measured variables. The number of observations was *n* = 17 for Figure 6a, and *n* = 21 for Figure 6b,c. Correlation coefficients (r) are represented by colored circles, with blue indicating negative correlations and red indicating positive correlations. Asterisks denote statistical significance (* *p* < 0.05, two-tailed Pearson correlation test). No multiple comparison correction was applied, as each analysis was restricted to a predefined set of variables. During 0 FT cycle, FSP binding, loading rate, and hydrophobic interactions showed significant negative correlations with RDS content (*p* < 0.05), indicating that higher FSP binding and stronger hydrophobic association corresponded to lower RDS (Figure 6a). C_ꝏ_ and eGI were also negatively correlated with FSP binding, suggesting that enhanced FSP-starch complexation was associated with reduced starch hydrolysis and lower glycemic response. For freeze-thawed samples, thermal stability and the retention of hydrogen bonds, hydrophobic interactions, and ionic bonds were significantly negatively correlated with digestive rate during 1 and 3 cycles (*p* < 0.05) (Figure 6b,c). Besides, we also observed that water permeability rate was positively correlated with RDS, hydrolysis equilibrium constant, and eGI (*p* < 0.05), indicating that poorer FT stability corresponded to higher digestion rate and glycemic index. These correlations indicated that FSP binding and FT stability were the dominant factors associated with the glycemic regulation performance of the complex during processing and storage.

### 3.6. Potential Mechanism

**Figure 7:**
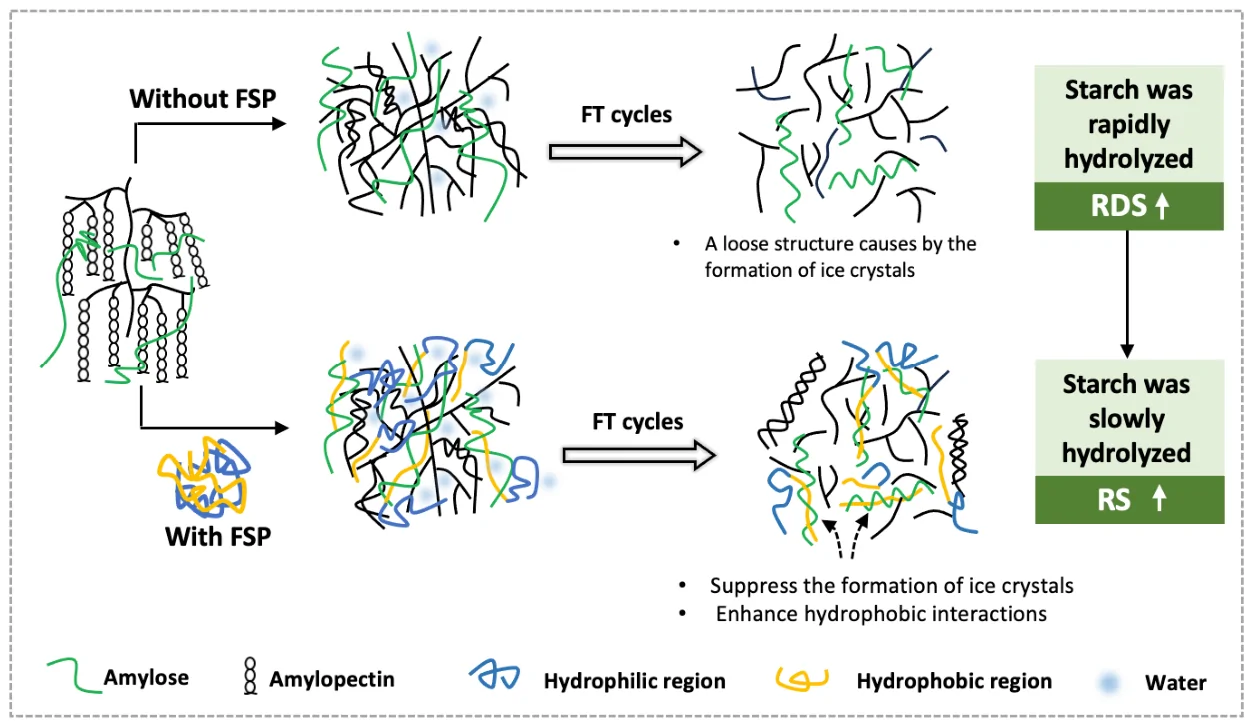
The possible mechanisms by which FSP affects starch digestion.

Without FSP, the starch gel network relies on disordered hydrogen bonds with large pores and abundant free water. FT cycles caused free water crystallization and expansion, damaging the network irreversibly, leading to gel collapse and amylose leaching. The amylose content of MS increased from 20.67% to 28.17%, while the average molecular weight remained unchanged, indicating physical dissociation rather than covalent bond breakage. This loose network exposes more enzyme action sites, accelerating starch hydrolysis and increasing blood glucose response (Figure 7). FSP was proposed to promote the formation of V-type structures. In this proposed mechanism, FSP bind with starch to form the complexes, densifying the gel network and enhancing water binding. This increased RS and limited hydrolysis. Meanwhile, FSP adsorbed onto ice crystal surfaces to inhibit crystal growth, synergizing with the network barrier. Consequently, amylose leaching was suppressed, and starch hydrolysis and AUC decrease.

Although studies have shown that other food components can enhance the FT stability of starch, such as caffeic acid which forms hydrogen bonds with the starch chains through phenolic hydroxyl groups, spatially hindering the close proximity and rearrangement of starch molecule chains during FT cycles, thereby increasing FT stability and increasing the content of RS [48]; guar gum formed an independent network structure in the starch gel, by increasing the viscosity of the system and filling the voids to delay water migration and ice crystal growth, but its RDS content instead increased from 38.35% to 41.74%, while the content of RS significantly decreased [47]. However, different from the above mechanisms, FSP inhibited recrystallization by specific interactions with the surface of ice crystals, and the formed the complex was relatively stable during FT cycles, which can effectively reduce post-meal blood glucose levels while maintaining the integrity of the starch crystalline structure, achieving the simultaneous consideration of FT protection and digestion regulation.

## 4. Conclusions

In conclusion, FSP effectively improved freeze-thaw stability of maize starch by inhibiting ice recrystallization and reducing permeability rate through hydrophobic-driven V-type complexation. Repeated FT cycles induced a destruction-reconstruction balance, where FSP promoted cold crystallization to compensate structural loss, as evidenced by DSC enthalpy rebound. FSP also protected starch from digestibility deterioration by limiting enzyme diffusion and increasing resistant starch, leading to reduced eGI and postprandial glucose responses in vivo. Correlation analysis validated that FSP binding and hydrophobic interactions were negatively correlated with RDS and eGI. This work demonstrated that FSP achieved triple functionality-antifreeze, water regulation, and digestive inhibition-through structural modification, providing theoretical guidance for applying fish-scale peptides in frozen starch-based foods to improve nutritional quality.

## Figures and Tables

**Table 1:** Changes of relaxation time of starchy samples during FT cycles.

T_2_ (ms)		MS	MS-2FSP	MS-4FSP	MS-6FSP	MS-8FSP	MS-10FSP
T_21_	0 Cycle	3.3 ± 2.1 ^aA^	1.47 ± 1.0 ^abA^	0.4 ± 0.1 ^bB^	0.6 ± 0.2 ^bA^	0.6 ± 0.3 ^bA^	0.6 ± 0.1 ^bB^
	1 Cycle	0.5 ± 0.1 ^bB^	0.65 ± 0.2 ^abA^	2.3 ± 1.1 ^abA^	0.9 ± 0.7 ^abA^	1.0 ± 1.0 ^abA^	9.2 ± 7.4 ^aA^
	3 Cycles	1.0 ± 0.8 ^aAB^	1.21 ± 0.8 ^aA^	0.9 ± 0.7 ^aAB^	0.6 ± 0.1 ^aA^	0.7 ± 0.27 ^aA^	0.8 ± 0.5 ^aAB^
T_22_	0 Cycle	22.0 ± 7.46 ^aB^	15.2 ± 4.2 ^aA^	11.5 ± 3.0 ^bB^	12.7 ± 2.63 ^bA^	19.47 ± 7.9 ^a bA^	10.4 ± 0.6 ^bC^
	1 Cycle	155.8 ± 91.7 ^aA^	41.1 ± 17.8 ^bA^	60.8 ± 32.5 ^aA^	24.4 ± 8.3 ^bA^	30.9 ± 4.8 ^bA^	77.5 ± 16.9 ^aA^
	3 Cycles	139.8 ± 128.7 ^aA^	321.2 ± 175.3 ^aB^	31.3 ± 6. 8 ^bA^	14.1 ± 8.3 ^cA^	24.5 ± 8.5 ^bcA^	32.4 ± 6.6 ^bB^
T_23_	0 Cycle	841.5 ± 52.6 ^aC^	761.8 ± 85.5 ^abB^	811.1 ± 0.0 ^aA^	682.5 ± 39.8 ^bA^	731. 9 ± 45.8 ^bA^	741.8 ± 120.1 ^abA^
	1 Cycle	1447.3 ± 484.5 ^aB^	479.2 ± 50.1 ^bC^	486.4 ± 77.5 ^bB^	450.0 ± 117.9 ^bcB^	355.9 ± 21.6 ^cC^	616.6 ± 60.8 ^bA^
	3 Cycles	2235.1 ± 139.7 ^aA^	1565.0 ± 667.1 ^aA^	396.2 ± 69.9 ^bB^	418.8 ± 26.2 ^bB^	506.9 ± 147.8 ^bB^	406.3 ± 46.2 ^bB^

Note: Different lowercase letters indicate significant differences among different FSP addition levels, and different uppercase letters indicate significant differences among different FT cycles (*p* < 0.05). The data were presented as the mean value ± SD, *n* = 3.

**Table 2:** BA (mg/g), LR (%), amylose content (%) and Mw (×10^6^ g/mol) of the complexes treated with 0, 1 and 3 FT cycles.

	Cycle	MS	MS-2FSP	MS-4FSP	MS-6FSP	MS-8FSP	MS-10FSP
BA	0	/	16.19 ± 2.89 ^eA^	33.20 ± 1.80 ^dA^	51.10 ± 2.75 ^cA^	64.56 ± 2.75 ^bA^	81.65 ± 2.89 ^aA^
	1	/	13.64 ± 2.27 ^eAB^	29.28 ± 2.18 ^dAB^	46.01 ± 2.27 ^cAB^	58.74 ± 6.81 ^bAB^	74.74 ± 4.41 ^aAB^
	3	/	11.83 ± 1.09 ^eB^	25.65 ± 2.27 ^dB^	43.46 ± 2.89 ^cB^	54.74 ± 1.67 ^bB^	71.10 ± 3.15 ^aB^
LR	0	/	80.95 ± 14.43 ^aA^	83.00 ± 4.50 ^aA^	85.17 ± 6.30 ^aA^	80.70 ± 3.43^aA^	81.65 ± 2.89 ^aA^
	1	/	68.22 ± 11.36 ^aA^	73.20 ± 5.45 ^aAB^	76.68 ± 3.79 ^aAB^	73.42 ± 8.52 ^aAB^	74.74 ± 4.41 ^aAB^
	3	/	59.13 ± 5.45 ^bB^	64.11 ± 5.68 ^abB^	72.44 ± 4.81 ^aB^	68.42 ± 2.08 ^aB^	71.10 ± 3.15 ^aB^
Amylose content	0	20.67 ± 2.75 ^aB^	20.00 ± 1.00 ^aB^	20.33 ± 2.47 ^aA^	21.00 ± 3.28 ^aA^	20.83 ± 1.53 ^aA^	21.33 ± 2.57 ^aA^
	1	22.67 ± 1.61 ^aB^	22.17 ± 1.89 ^aAB^	21.50 ± 1.00 ^aA^	23.33 ± 1.53 ^aA^	22.00 ± 1.80 ^aA^	22.50 ± 1.32 ^aA^
	3	28.17 ± 2.25^aA^	26.33 ± 2.25 ^abA^	23.83 ± 1.61 ^bA^	24.50 ± 1.80 ^abA^	24.17 ± 1.53 ^abA^	24.50 ± 1.73 ^abA^
M_w_	0	2.13 ± 0.31 ^aA^	2.19 ± 0.21 ^aA^	2.14 ± 0.28 ^aA^	2.05 ± 0.47 ^aA^	2.13 ± 0.24 ^aA^	2.06 ± 0.26 ^aA^
	1	2.04 ± 0.18 ^aA^	2.14 ± 0.23 ^aA^	2.09 ± 0.30 ^aA^	2.11 ± 0.35 ^aA^	2.22 ± 0.21 ^aA^	2.15 ± 0.27 ^aA^
	3	2.01 ± 0.14 ^aA^	2.10 ± 0.27 ^aA^	2.07 ± 0.19 ^aA^	2.06 ± 0.29 ^aA^	2.16 ± 0.12 ^aA^	2.22 ± 0.11 ^aA^

Note: Different lowercase letters indicated significant differences among different FSP addition levels, and different uppercase letters indicated significant differences among different FT cycles (*p* < 0.05). The data were presented as the mean value ± SD, *n* = 3.

**Table 3:** Thermal parameters of the starchy samples.

	Peak I				Peak II			
Samples	To (°C)	Tp (°C)	Tc (°C)	ΔH (J/g)	To (°C)	Tp (°C)	Tc (°C)	ΔH (J/g)
MS-0	--	--	--	--	96.6 ± 0.7 ^abB^	106.2 ± 0.8 ^aA^	112.3 ± 1.1 ^aB^	0.4 ± 0.0 ^cA^
MS-2FSP-0	--	--	--	--	98.3 ± 0.6 ^aA^	106.3 ± 0.6 ^aA^	112.3 ± 0. 5 ^aA^	0.6 ± 0.0 ^bA^
MS-4FSP-0	--	--	--	--	96.5 ± 0.6 ^bA^	104.8 ± 0.4 ^bA^	111.7 ± 0. 8 ^aA^	0.4 ± 0.0 ^cB^
MS-6FSP-0	--	--	--	--	98.2 ± 0. 5 ^aA^	104.3 ± 0.3 ^bA^	110.6 ± 0.3 ^bB^	0.4 ± 0.0 ^dB^
MS-8FSP-0	--	--	--	--	97.6 ± 0. 4 ^abA^	106.7 ± 0.7 ^aA^	112.7 ± 0.4 ^aA^	0.6 ± 0.0 ^bA^
MS-10FSP-0	--	--	--	--	94.6 ± 1.2 ^bA^	106.9 ± 0.5 ^aA^	111.4 ± 0.5 ^abA^	0.8 ± 0.0 ^aA^
MS-1	42.7 ± 1.2 ^cB^	51.0 ± 1.3 ^bB^	61.2 ± 1.3 ^bB^	2.6 ± 0.2 ^dA^	100.6 ± 0.3 ^aA^	103.1 ± 0.2 ^bB^	113.1 ± 0.6 ^aB^	0.0 ± 0.0 ^dC^
MS-2FSP-1	45.1 ± 0.3 ^bA^	56.1 ± 2.3 ^aA^	64.7 ± 0.4 ^aA^	3.5 ± 0.1 ^bcB^	84.9 ± 0.8 ^cC^	94.5 ± 0.4 ^dB^	101.9 ± 0.6 ^eB^	0.2 ± 0.0 ^eC^
MS-4FSP-1	46.9 ± 0.8 ^aA^	54.9 ± 0.7 ^aA^	63.6 ± 0.5 ^aA^	3.2 ± 0.1 ^cB^	93.9 ± 0.7 ^bC^	103.7 ± 1.0 ^abA^	110.0 ± 0.5 ^cC^	0.5 ± 0.0 ^cA^
MS-6FSP-1	46.9 ± 0.3 ^aA^	54.8 ± 0.3 ^aA^	64.3 ± 0.4 ^aA^	3.4 ± 0.1 ^bB^	92.1 ± 0.5 ^bB^	100.8 ± 0.5 ^cC^	105.4 ± 0.9 ^cC^	0.3 ± 0.1 ^dC^
MS-8FSP-1	46.9 ± 0.3 ^aA^	55.3 ± 0.6 ^aA^	64.1 ± 0.6 ^aA^	4.6 ± 0.3 ^aB^	92.6 ± 0.5 ^bB^	104.3 ± 0.5 ^aB^	111.0 ± 0.4 ^dC^	0.5 ± 0.0 ^bB^
MS-10FSP-1	46.7 ± 0.5 ^aA^	55.0 ± 0.3 ^aA^	63.1 ± 0.3 ^abA^	4.5 ± 0.1 ^aB^	93.2 ± 0.3 ^bA^	100.8 ± 0.6 ^cB^	112.2 ± 0.3 ^bA^	0.6 ± 0.0 ^aB^
MS-3	45.5 ± 0.7 ^bcA^	54.7 ± 0.7 ^aA^	63.6 ± 0.6 ^abA^	2.8 ± 0.2 ^cA^	94.7 ± 0.3 ^bC^	101.7 ± 0.3 ^cC^	115.5 ± 0.4 ^aA^	0.2 ± 0.0 ^cB^
MS-2FSP-3	44.8 ± 0.5 ^cA^	54.7 ± 0.40^aA^	63.4 ± 1.0 ^abA^	4.6 ± 0.1 ^bA^	95.1 ± 0.3 ^bB^	105.2 ± 0.6 ^aA^	113.2 ± 0.5 ^bcA^	0.5 ± 0.0 ^bB^
MS-4FSP-3	45.9 ± 0.4 ^bA^	54.7 ± 0.3 ^aA^	63.6 ± 0.6 ^abA^	5.0 ± 0.0 ^aA^	94.4 ± 0.5^bcB^	105.9 ± 0.5 ^aA^	114.2 ± 0.5 ^bB^	0.6 ± 0.1 ^aA^
MS-6FSP-3	46.9 ± 0.3 ^abA^	55.3 ± 0.7 ^a^	63.6 ± 0.5 ^abA^	4.7 ± 0.1 ^bA^	91.7 ± 0.5 ^dB^	103.6 ± 0.8 ^bB^	112.7 ± 0.8 ^cA^	0.6 ± 0.0 ^aA^
MS-8FSP-3	46.4 ± 0. 9 ^abA^	54.8 ± 0.4 ^aA^	64.2 ± 0.5 ^aA^	5.1 ± 0.0 ^aA^	98.6 ± 0.4 ^aA^	106.2 ± 0.7 ^aA^	113.9 ± 0.2 ^bA^	0.5 ± 0.1 ^bAB^
MS-10FSP-3	47.0 ± 0.5 ^aA^	54.8 ± 0.5 ^aA^	63.1 ± 0.3 ^bA^	5.0 ± 0.2 ^aA^	93.1 ± 0.8 ^cA^	106.5 ± 0.7 ^aA^	112.4 ± 0.7 ^cA^	0.5 ± 0.1 ^abB^

Note: Different lowercase letters indicated significant differences among different FSP addition levels, and different uppercase letters indicated significant differences among different FT cycles (*p* < 0.05). The data were presented as the mean value ± SD, *n* = 3.

**Table 4:** Digestive parameters during in vitro digestion.

Sample	0 Cycle		1 Cycle		3 Cycle	
	C∞ (%)	eGI	C∞ (%)	eGI	C∞ (%)	eGI
MS	82.67 ± 3.41 ^aB^	70.75 ± 2.12 ^aB^	87.77 ± 0.55 ^aA^	76.83 ± 0.71 ^aA^	88.13 ± 1.69 ^aA^	78.20 ± 1.86 ^abA^
MS-2FSP	71.46 ± 0.41 ^bB^	69.79 ± 0.35 ^aB^	79.74 ± 4.44 ^bA^	76.93 ± 3.83 ^aA^	82.77 ± 0.71 ^bA^	79.54 ± 0.61 ^aA^
MS-4FSP	64.02 ± 0.55 ^cB^	63.38 ± 0.48 ^bC^	73.41 ± 0.25 ^bcA^	71.47 ± 0.22 ^bB^	77.88 ± 3.28 ^bA^	75.33 ± 2.82 ^bA^
MS-6FSP	59.43 ± 0.61 ^cdB^	59.42 ± 0.53 ^cB^	68.59 ± 6.66 ^bcA^	67.32 ± 5.74 ^bA^	71.16 ± 4.35 ^cA^	69.54 ± 3.75 ^bcA^
MS-8FSP	59.47 ± 0.99 ^dB^	59.46 ± 0.85 ^cA^	62.09 ± 6.42 ^cdA^	61.71 ± 5.54 ^ccA^	64.24 ± 2.76 ^cA^	63.57 ± 2.38 ^cA^
MS-10FSP	57.07 ± 1.01 ^dB^	57.39 ± 0.88 ^dB^	57.76 ± 3.51 ^dB^	57.99 ± 3.02 ^cB^	68.85 ± 3.23 ^cA^	67.54 ± 2.78 ^cA^

Note: Different lowercase letters indicated significant differences among different FSP addition levels, and different uppercase letters indicated significant differences among different FT cycles (*p* < 0.05). The data were presented as the mean value ± SD, *n* = 3.

## Data Availability

The original contributions presented in this study are included in the article/Supplementary Materials. Further inquiries can be directed to the corresponding authors.

## Supplementary Materials

The following supporting information can be downloaded at: https://www.mdpi.com/article/10.3390/foods15213943/s1, S1. Molecular weight distribution, Table S1: Molecular weight distribution of FSP; S2. Amino Acid Composition, Table S2: Amino Acid Composition of FSP (units: g/100 g); Table S3: RC (%) of the complexes treated with 0, 1 and 3 FT cycles.
