# Supplementary material for: Reduced Digestibility of Maize Starch in Freeze-Thaw Cycles: Mechanistic Insights into the Role of Fish Scale Peptides
Source: Foods. 2026 Nov 3;15(21):3943. doi: 10.3390/foods15213943 (PMC13763033; doi:10.3390/foods15213943)
Supplement: Supplementary file 1 [file foods-15-03943-s001.zip › foods-4602523-supplementary.pdf]

The prepared method of FSP

The preparation process of FSP is as follows: Using the fish scales of red snapper as the raw material, after washing, drying, purification and grinding, they are dispersed in deionized water at a concentration of 6% (w/w). First, they are subjected to ultrasonic treatment at 50°C for 1.5 h, then subjected to high-temperature and high-pressure treatment at 121°C and 0.1 MPa for 1 h to release the collagen from the fish scales; subsequently, the pH of the mixture is adjusted to 5.8, and 9.6% (w/w) papain is added for enzymatic hydrolysis at 60°C for 7.8 h; after the enzymatic hydrolysis is completed, the proteinase is inactivated at 100°C for 10 min, then centrifuged at 10,000 rpm for 10 min, and the supernatant is freeze-dried to obtain FSP.

S1. Molecular weight distribution

Determination method: HPLC (Waters™ 650E Advanced Protein Purification System)

Table S1 Molecular weight distribution of FSP

Molecular weight distribution	Proportion
< 180 Da	36.61%
180–1000 Da	58.00%
1000–3000 Da	4.46%
> 3000 Da	0.93%

S2. Amino Acid Composition

Determination method: High-speed amino acid analyzer (Hitachi L-8900). The samples were hydrolyzed with 6 M HCl at 110°C for 24 hours before measurement.

Table S2 Amino Acid Composition of FSP (units: g/100 g)

Amino Acid	Composition	Amino Acid	Composition
Asp	4.51	Cys	2.11
Glu	8.74	Val	2.53
Ser	2.45	Met	1.90

His	3.62	Phe	2.01
Gly	19.20	Ile	1.27
Thr	1.88	Leu	2.24
Arg	5.94	Lys	2.97
Ala	8.18	Pro	11.78
Tyr	1.12		

Table S3 RC (%) of the complexes treated with 0, 1 and 3 FT cycles.

	Cycle	MS	MS-2FSP	MS-4FSP	MS-6FSP	MS-8FSP	MS-10FSP
	**0**	5.13	0.29	4.36	2.37	6.13	5.05
**RC**	**1**	4.79	1.06	4.52	1.23	3.68	4.05
	**3**	2.37	0.12	4.01	0.56	5.29	0.61
